# Non-invasive *in vivo* imaging of UCP1 expression in live mice via near-infrared fluorescent protein iRFP720

**DOI:** 10.1371/journal.pone.0225213

**Published:** 2019-11-15

**Authors:** Aya Fukuda, Shiho Honda, Norie Fujioka, Yuya Sekiguchi, Seiya Mizuno, Yoshihiro Miwa, Fumihiro Sugiyama, Yohei Hayashi, Ken Nishimura, Koji Hisatake

**Affiliations:** 1 Laboratory of Gene Regulation, University of Tsukuba, Tsukuba, Ibaraki, Japan; 2 Laboratory of Animal Science, University of Tsukuba, Tsukuba, Ibaraki, Japan; 3 Laboratory of Anatomy and Embryology, University of Tsukuba, Tsukuba, Ibaraki, Japan; University of Texas Health Science Center at Houston, UNITED STATES

## Abstract

Uncoupling protein 1 (UCP1) is a mitochondrial protein that is expressed in both brown and beige adipocytes. UCP1 uncouples the mitochondrial electron transport chain from ATP synthesis to produce heat via non-shivering thermogenesis. Due to their ability to dissipate energy as heat and ameliorate metabolic disorders, UCP1-expressing adipocytes are considered as a potential target for anti-obesity treatment. To monitor the expression of UCP1 in live mice in a non-invasive manner, we generated the *Ucp1-iRFP720* knock-in (*Ucp1-iRFP720* KI) mice, in which the gene encoding a near-infrared fluorescent protein iRFP720 is inserted into the *Ucp1* gene locus. Using the heterozygous *Ucp1-iRFP720* KI mice, we observed robust iRFP fluorescence in the interscapular region where brown adipose tissue is located. Moreover, the iRFP fluorescence was clearly observable in inguinal white adipose tissues in live mice administered with β3-adrenergic receptor agonist CL316,243. We also found that the homozygous *Ucp1-iRFP720* KI mice, which are deficient in UCP1, displayed prominent iRFP fluorescence in the inguinal regions at the standard housing temperature. Consistent with this, the mice exhibited expanded populations of beige-like adipocytes in inguinal white adipose tissue, in which the *Ucp1* promoter was dramatically activated. Thus, the *Ucp1-iRFP720* KI mice provide a convenient model for non-invasive *in vivo* imaging of UCP1 expression in both brown and beige adipocytes in live mice.

## Introduction

Uncoupling protein 1 (UCP1) is a mitochondrial protein that uncouples respiration from ATP synthesis to produce heat [1]. UCP1 is expressed in brown adipose tissue (BAT) as well as in some white adipose tissues (WATs), in which beige adipocytes are induced upon various stimuli [2]. Recent studies revealed that human adults possess active BAT [3][4][5], which appears to comprise both classical brown adipocytes and beige adipocytes [6][7][8][9]. Human BAT is associated with leanness [3][4][10], and its reduction during aging may accelerate accumulation of body fat [11]. Human BAT may play a protective role against hyperglycemia and related metabolic disorders [12], and, if activated by cold exposure, increases energy dissipation, reduces fat mass, and improves insulin sensitivity [13][14][15][16].

Owing to their anti-obesity potential, brown and beige adipocytes may be manipulated to reduce body weight and ameliorate metabolic disorders [17][18][19]. Beige adipocytes, in particular, are promising targets for treating obesity and its related disorders because of their inducibility in WAT, which is abundant in obese patients. Indeed, when beige adipocytes are ablated by adipocyte-specific knockout of PRDM16, mice develop high-fat diet-induced obesity and insulin resistance [20]. In rodents, beige adipocytes are induced by a number of stimuli, which include cold exposure, thiazolidinediones (peroxisome proliferator-activated receptor gamma [PPAR-γ] agonists) [21], β3-adrenergic receptor agonists [22], or physical exercise [23].

Like classical brown adipocytes, beige adipocytes clearly depend upon UCP1 for thermogenesis in both mice and humans [24][25]. However, studies on UCP1-deficient mice revealed the presence of alternate thermogenesis, which is independent of UCP1 [26][27]. Recent studies on beige adipocytes uncovered mechanisms of alternate thermogenesis, such as creatine-dependent ADP/ATP substrate cycling [28][29][30] and calcium cycling [31], both of which are futile cycles in cellular metabolism that dissipate heat. Thus, beige adipocytes have multiple thermogenic mechanisms that could potentially be targeted and manipulated by drugs. Therefore, *in vivo* imaging of beige adipocytes could be useful in identifying physiological conditions that induce beige adipocytes, dissecting the molecular mechanisms of beige adipocyte induction, and testing drugs for anti-obesity treatment.

Imaging of biological processes in live mice has been greatly facilitated by the recent development of near-infrared (NIR) fluorescent proteins [32], which are now widely used for *in vivo* imaging. NIR fluorescent proteins possess red-shifted absorption spectra that range from 670 to 720 nm, and thereby suffer from relatively low absorption by biological components. To emit fluorescence, bacterial phytochrome-based NIR fluorescent proteins require biliverdin, which typically needs to be supplied externally. However, iRFPs, which were engineered to emit fluorescence at the level of endogenous biliverdin in cells, no longer require an external supply of biliverdin [33]. Among the five spectrally distinct iRFPs (iRFP670, iRFP682, iRFP702, iRFP713, and iRFP720), iRFP720 is the most red-shifted NIR fluorescent protein, and presumably best suited for *in vivo* tissue imaging in live mice [34][35].

Here we used CRISPR/Cas9-based genome editing to generate the *Ucp1-iRFP720* knock-in (*Ucp1-iRFP720* KI) mice by inserting the *iRFP720* gene into the *Ucp1* locus and simultaneously inactivating the *Ucp1* gene. The mice express UCP1 and iRFP720 under the control of the *Ucp1* promoter at its endogenous locus, without any extra amino acids added at their ends. The heterozygous *Ucp1-iRFP720* KI mice allowed *in vivo* imaging of UCP1-expressing brown adipocytes as well as beige adipocytes induced by a β3-adrenergic receptor agonist, CL316,243. The homozygous *Ucp1-iRFP720* KI mice showed increased expression of iRFP720 in inguinal WAT (iWAT) when housed at the standard ambient temperature. The *Ucp1* promoter activity in the homozygous *Ucp1-iRFP720* KI mice is significantly increased in iWAT, which contained clusters of small adipocytes morphologically similar to beige adipocytes. These results indicate that heterozygous and homozygous *Ucp1-iRFP720* KI mice may serve as an excellent tool for *in vivo* imaging of beige adipocyte induction in live mice under various environmental and nutritional conditions.

## Materials and methods

### Construction of vectors for genome editing

Guide RNA candidates were designed using CRISPR direct software (https://crispr.dbcls.jp) [36]. To construct guide RNA-expression vectors, four pairs of oligonucleotides (5 pmoles each) encoding the guide RNAs were denatured in the buffer containing 10 mM Tris-HCl (pH8.0), 100 mM NaCl and 1 mM ethylenediaminetetraacetic acid (EDTA) at 95°C for 4 minutes and were annealed by gradually cooling to room temperature. The annealed oligonucleotide pairs were individually inserted into the *Bbs*I site of the pX330-U6-Chimeric_BB-CBh-hSp9 vector (Addgene 42230). To construct the donor vector, the mouse *Ucp1* gene fragments were prepared by PCR from the BAC clone (pBACe3.6-B6Ng01-162F24, RIKEN RDB07573). Then, the DNA fragment containing the mouse *Ucp1* gene (-3.87K to +248 (upstream of ATG)), an artificial intron, the *iRFP720* gene, the bovine growth hormone polyadenylation signal (BGHpA) and the mouse *Ucp1* gene (+249 to +4.56K) were inserted sequentially into pBluescript II KS (-) vector. The artificial intron, which is a chimera of *β-globin* and *IgG* introns, was chemically synthesized. All the constructed plasmids were sequenced entirely to rule out spurious mutations.

### *In vitro* assay for evaluating the gRNA/Cas9-mediated homologous recombination

The mouse *Ucp1* gene fragment from +1 to +500, which contains the target sequences of the gRNAs, was inserted into the *Bam*HI site of pCAG-EGxxFP vector (Addgene 50716). Human embryonic kidney (HEK293T) cells were seeded in a 24-well plate at 2x10^5^ cells/well one day before transfection and incubated at 37°C in a 5% CO_2_ incubator. Each gRNA/Cas9-expression vector (0.5 μg), together with the pCAG-EGxxFP-Ucp1(+1 ~ +500) vector (0.5 μg), was transfected into the cells using Lipofectamine LTX (Thermo Fisher Scientific Inc.), and the fluorescence signal was observed two days after transfection.

### Generation of the *Ucp1-iRFP720* knock-in mice and genomic PCR analyses

The gRNA2/Cas9 expression vector and the donor vector were injected into the pronuclei of 248 fertilized C57BL/6J mouse (Charles River Laboratories Japan, Inc.) eggs, and 236 embryos were transferred into the oviduct of pseudo-pregnant mice. Eighty-two live pups (F0) were obtained, and genome DNA was prepared from the tail of each F0 mouse and analyzed by PCR for the presence of the integrated *iRFP720* gene. To detect the *iRFP720* gene, 5 ng each of the genome DNA was analyzed by PCR in a 5 μl reactions containing 2.5 μl of AmpliTaq Gold 360 Master Mix (ThermoFisher Scientific Inc.) and 1.5 pmoles each of the primers. To confirm the correct insertion of the *iRFP720* gene at the *Ucp1* locus, 10 ng each of the genome DNA was analyzed by PCR using 0.25 unit of PrimeStar GXL DNA Polymerase (TaKaRa Bio Inc.) and 3 pmoles each of the primers in a 10 μl reaction. In addition, 20 ng of genome DNA was analyzed by PCR containing 0.1 unit of KOD FX Neo (TOYOBO CO., LTD.) and 1.5 pmoles each of the primers in a 5 μl reaction. To determine the number of the alleles harboring the *iRFP720* gene, 20 ng of the genome DNA was analyzed by PCR containing 0.2 unit of KOD FX Neo and 1.5 pmoles each of the primers in a 5 μl reaction. All the PCR reactions were performed according to the manufactures’ instructions, and the sequences of primers are shown in the S1 Table. Genome DNA from a wild-type (WT) mouse was used as a negative control, and a DNA fragment that spans the upstream and downstream regions of the *iRFP720*-knock-in *Ucp1* gene, including all primer target sites, was used as a positive control.

### *In vivo* imaging of the *Ucp1-iRFP720* knock-in mice

Mice (male, 16~20 weeks old) were housed at 22–24°C on a 10hr/14hr dark-light cycle, with free access to water and food. Mice were routinely fed standard chow (MF, Oriental Yeast Co., Ltd.), which were replaced with low-fluorescence chow (iVid#2, Oriental Yeast Co., Ltd.) one week before observation of fluorescence by IVIS. Fluorescence images of the *Ucp1-iRFP720* knock-in mice were captured and analyzed by IVIS Spectrum imaging system (Summit Pharmaceuticals International Corp.). Quantification of the fluorescence was done by the software Living Image® 4.5.2. (Summit Pharmaceuticals International Corp.). To detect iRFP fluorescence *in vivo*, the hair was removed in the dorsal and inguinal areas of mice, and the mice were anesthetized by 2.5–3.0% isoflurane during observation of the iRFP720-derived fluorescence (excitation: 675 nm, emission: 720 nm). To detect iRFP fluorescence *ex vivo*, BAT and WAT were dissected from mice, which were sacrificed by cervical dislocation under isoflurane anesthesia, and observed directly by IVIS Spectrum imaging system. All mouse experiments were prospectively approved by the ethics committee for Animal Experiments of the University of Tsukuba (Approval number: 17–099, 17–397, 18–026).

### Western blot analysis

Adipose tissues were minced, and the proteins were extracted by sonication in RIPA buffer [50 mM Tris-HCl (pH 8.0), 150 mM NaCl, 2 mM EDTA (pH 8.0), 1% NP-40, 0.5% sodium deoxycholate, 0.1% sodium dodecyl sulfate (SDS)] containing protease inhibitor cocktail and 0.5 mM phenylmethylsulfonyl fluoride (PMSF). One microgram (BAT) or 7.5 μg (iWAT) of protein from each cell extract was used for SDS-PAGE, followed by western blotting using the primary antibody against UCP1 (Abcam, ab10983, 1:2000) and the horseradish peroxidase-labeled secondary antibody against rabbit IgG (GE healthcare, NA934V, 1:3000). As a loading control, GAPDH was detected by the primary antibody against GAPDH (Santa Cruz Biotechnology Inc., sc-32233, 1:200) and the horseradish peroxidase-labeled secondary antibody against mouse IgG (GE healthcare, LNA931V/AG, 1:3000). The intensity for UCP1 was normalized by that for GAPDH, and the relative intensity to wild type was calculated.

### Hematoxylin and eosin (HE) staining

Adipose tissues were isolated from the mice and soaked in neutral buffered formalin for several days. The tissues were then paraffin-embedded, sectioned, mounted onto slides and stained with hematoxylin and eosin at the Medical Laboratory for Tissue Sample Preparation at the University of Tsukuba according to the standard protocol.

### Total RNA preparation and quantitative RT-PCR analysis

Total RNA was prepared from the minced adipose tissues using Sepasol-RNA I Super G (Nacalai Tesque, Inc.), and the complementary DNA (cDNA) was prepared using SuperScript III reverse transcriptase (ThermoFisher Scientific Inc.) according to the manufactures’ instructions. Using the cDNA, quantitative PCR was performed using the primer sets shown in the table S2 Table. The mRNA levels were normalized to the values of *Nono* (Non-POU domain-containing octamer-binding protein) [37], and the relative amounts of the mRNA were calculated using the the 2^−ΔΔCt^ method.

## Results

### Generation of *Ucp1-iRFP720* Knock-In (*Ucp1-iRFP720* KI) mice

**Fig 1:**
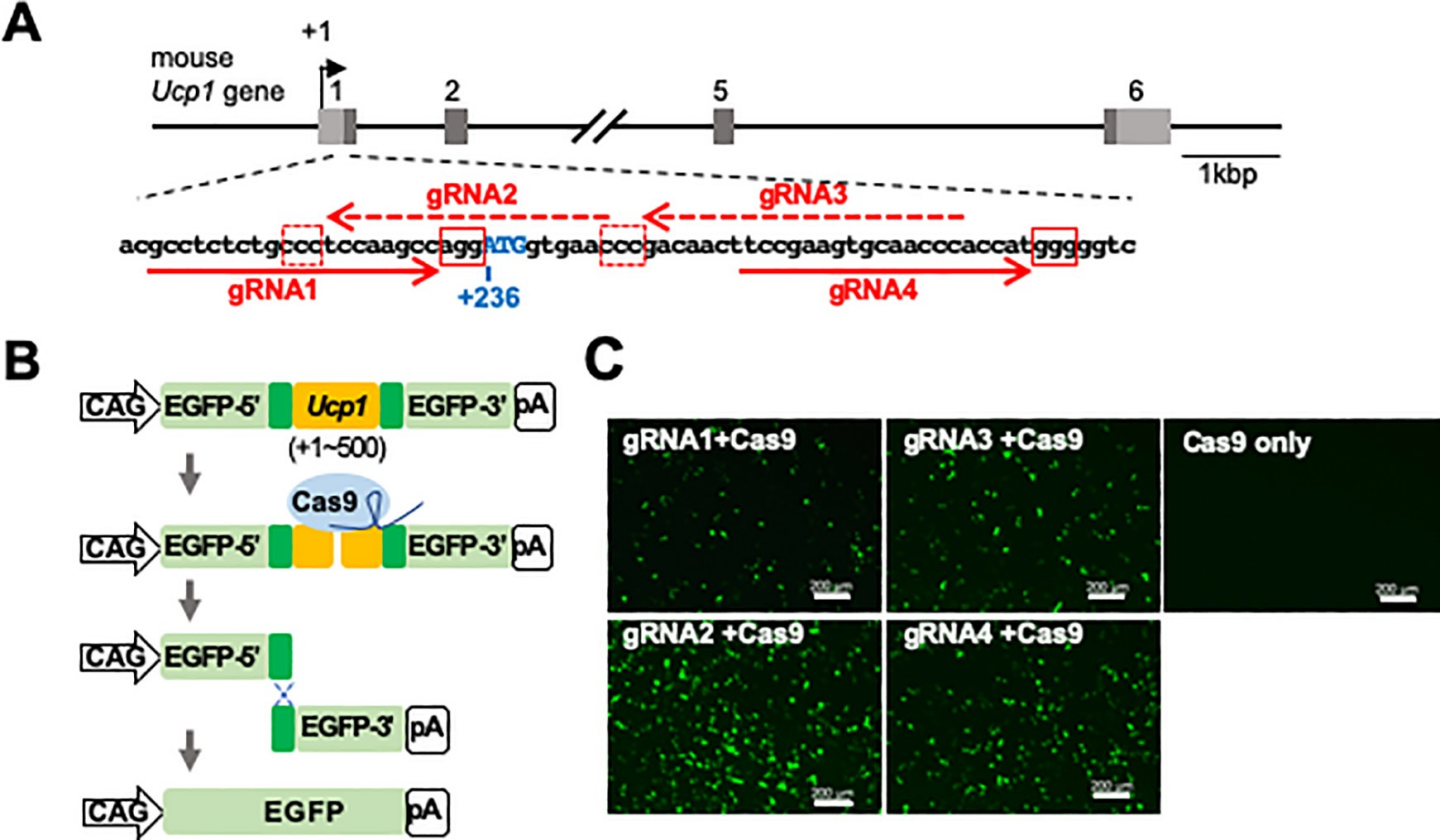
Selection of the optimum gRNA sequence to generate *Ucp1-iRFP720* KI mice. (A) Structure of the mouse *Ucp1* gene. The boxes and the numbers above each box indicate the exons of the mouse *Ucp1* gene. Light and dark gray regions within the exons indicate untranslated and translated regions, respectively. The position of the transcription start site is indicated by +1. The DNA sequence around the translation start site of the mouse *Ucp1* gene is shown. The red arrows indicate the target sequences of the gRNAs (dashed arrows indicate the complementary sequences). The red boxes show the protospacer adjacent motif (PAM) sequences. (B) The assay system for estimating the functional genome editing efficiency of gRNAs. The *Ucp1* gene (+1 ~ +500) containing the gRNA target sites (shown in orange) was inserted into pCAG-EGxxFP vector. When recruited by the gRNA (shown in dark blue) to the target site, the Cas9 nuclease (shown in light blue) introduces a double strand break in the target DNA. Because of homologous recombination between the duplicated sequences (shown in dark green), the EGFP-coding gene is recovered. (C) Fluorescence image of HEK293T cells two days after transfection. The Cas9 nuclease was expressed together with one of the four gRNA candidates (gRNA1~gRNA4) or without gRNA (Cas9 only). Scale bar, 200 μm.

To non-invasively visualize the expression of UCP1 in live mice, we inserted the gene encoding *iRFP720*, a near-infrared fluorescent protein, into the mouse *Ucp1* locus using the CRISPR/Cas9-mediated homologous recombination. Four guide RNAs (gRNAs) were designed around the translation initiation site of the *Ucp1* gene (Fig 1A) and tested for the efficiency of double-strand DNA break repair by homologous recombination using the previously reported assay system (Fig 1B) [38]. Among the four tested gRNAs, gRNA2 showed the highest number of EGFP signals in cells when co-expressed with Cas9 (Fig 1C). No EGFP signal was observed without gRNA (Fig 1C, Cas9 only), suggesting that the EGFP signals were derived from the gRNA-induced double-strand DNA break and homologous recombination. These results indicate that gRNA2 induces Cas9-mediated double-strand DNA breaks and homologous recombination with the highest efficiency among tested gRNAs.

**Fig 2:**
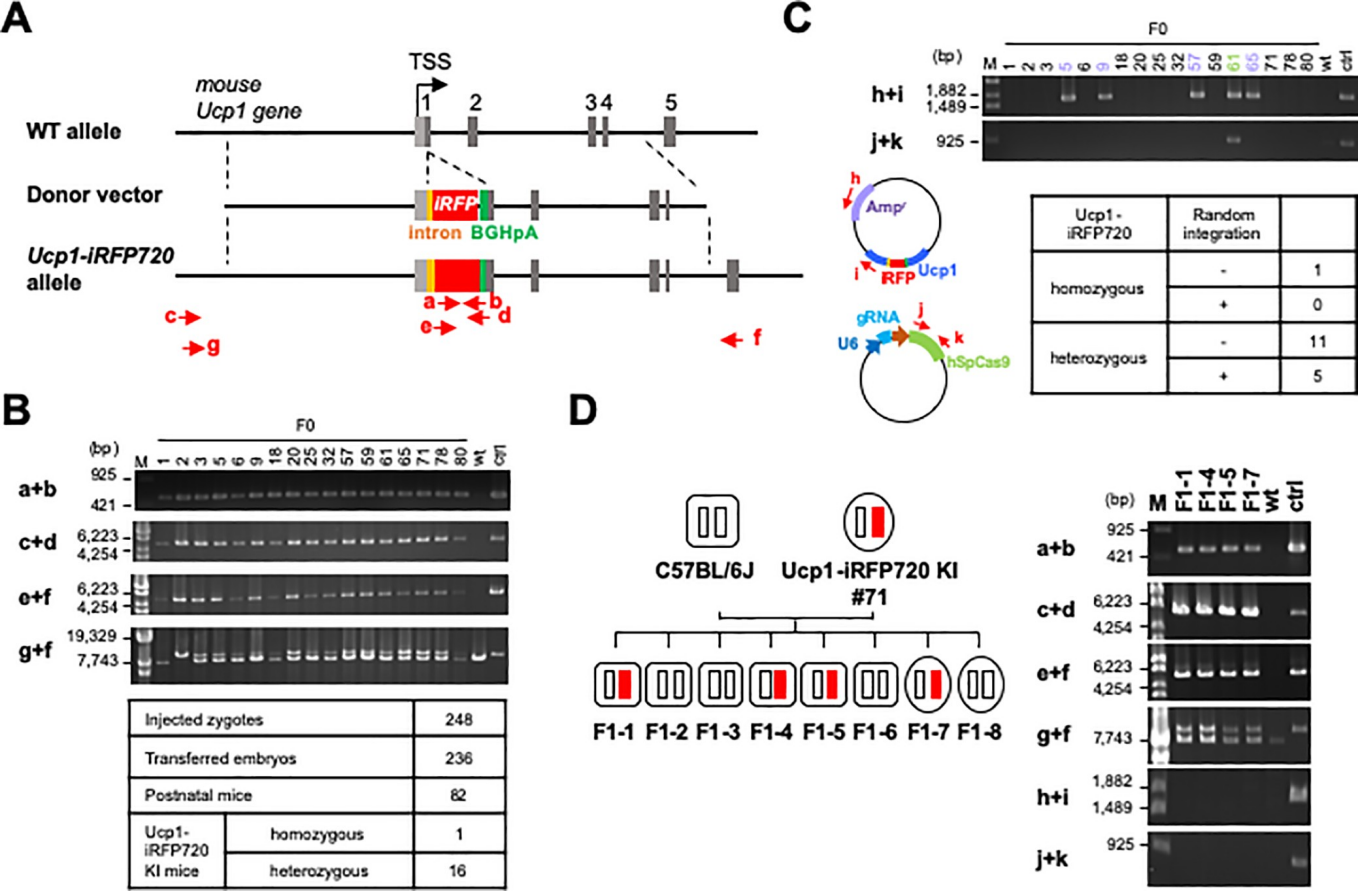
Generation of *Ucp1-iRFP720* KI mice. (A) Targeting of the *iRFP720* gene into the *Ucp1* locus. The donor vector contains an artificial intron (orange box), *iRFP720* gene (red box) and the bovine growth factor gene-derived polyadenylation signal (BGHpA) (green box). Structure of the targeted allele is indicated together with the primers used for genomic PCR (red arrows). (B) Genomic PCR analysis of F0 mice carrying the *Ucp1-iRFP720* KI allele. M, marker; wt, wild-type; ctrl, positive control (a DNA fragment that covers the amplicon sequence between the primers c and f in Fig 2A). (C) PCR analysis of the donor vector and the gRNA/Cas9-expression vector randomly integrated into the F0 mouse genome. The table shows the summary of random integration in the F0 mouse genome carrying the *Ucp1-iRFP720* allele. (D) Mendelian inheritance of the *Ucp1-iRFP720* KI allele. One of the *Ucp1-iRFP720* KI mice (#71) was crossed with a WT mouse, and eight F1 mice were born. Their genotypes were analyzed by PCR using the primer sets shown in Fig 2A. Small white and red rectangles show WT *Ucp1* and *Ucp1-iRFP720* KI alleles, respectively. Rounded rectangles and ellipses indicate male and female mice, respectively.

We then constructed the donor vector, that includes the *iRFP720* gene and the bovine growth hormone polyadenylation signal (BGHpA) inserted at the translation start site of the *Ucp1* gene (Fig 2A). The *iRFP720* gene was inserted at the initiation codon of the *Ucp1* gene such that the initiation codon of UCP1 is used for translation of iRFP720. Although the *Ucp1* gene in the knock-in allele will be disrupted, use of the 2A sequence was avoided to prevent altering the functions of iRFP720 and UCP1. In addition, an intron fragment was inserted into the upstream region of the *iRFP720* gene to enhance its expression [39][40]. After injection of the gRNA2/Cas9-expression vector and donor vector into fertilized mouse eggs, 236 embryos were transferred to the oviduct of pseudo-pregnant mice, which delivered 82 postnatal founder mice (F0). Genomic PCR analyses of the F0 mice revealed that 17 mice had the *iRFP720* gene inserted at the *Ucp1* locus (Fig 2B, a+b, c+d, e+f). Although most *iRFP720*-positive mice were heterozygous for the knock-in allele, one mouse (F0-2) was homozygous (Fig 2B, g+f). As summarized in the table, the *iRFP720* gene was successfully knocked-in at the *Ucp1* locus in ~20% (17 out of 82) of the F0 mice. To exclude the presence of randomly integrated vectors, we analyzed the genome of the *iRFP720*-positive mice using PCR with primer sets specific for the donor vector or the gRNA2/Cas9-expression vector. As shown in Fig 2C, five out of seventeen *iRFP720*-positive mice (F0-5, 9, 57, 61 and 65) exhibited the donor vector (h+i) integrated in their genome, and one of the mice (F0-61) had the integrated gRNA/Cas9-expression vector as well (j+k). In conclusion, we obtained 12 lines (1 homozygous mouse and 11 heterozygous mice) of the *Ucp1-iRFP720* KI F0 mice, C57BL/6J-*Ucp1*^em1(iRFP720)Utgr^, which were free of randomly integrated vectors (Fig 2C).

We then confirmed the inheritance of the *iRFP720* transgene to the next generation by crossing F0 mice with WT C57BL/6J mice. Given the numbers of F1 pups in a litter, the *iRFP720* gene knocked-in at the *Ucp1* locus did not appear to affect fertility. In addition, genomic PCR analysis revealed that the F1 mice inherited the *iRFP720* gene at an expected frequency (Fig 2D, a+b, c+d, e+f, g+f), without any random integration of the vectors (Fig 2D, h+i, j+k). These results indicate that the *iRFP720* gene of the *Ucp1-iRFP720* KI mice is inherited in a Mendelian fashion.

### *In vivo* imaging of BAT in *Ucp1-iRFP720* KI mice

**Fig 3:**
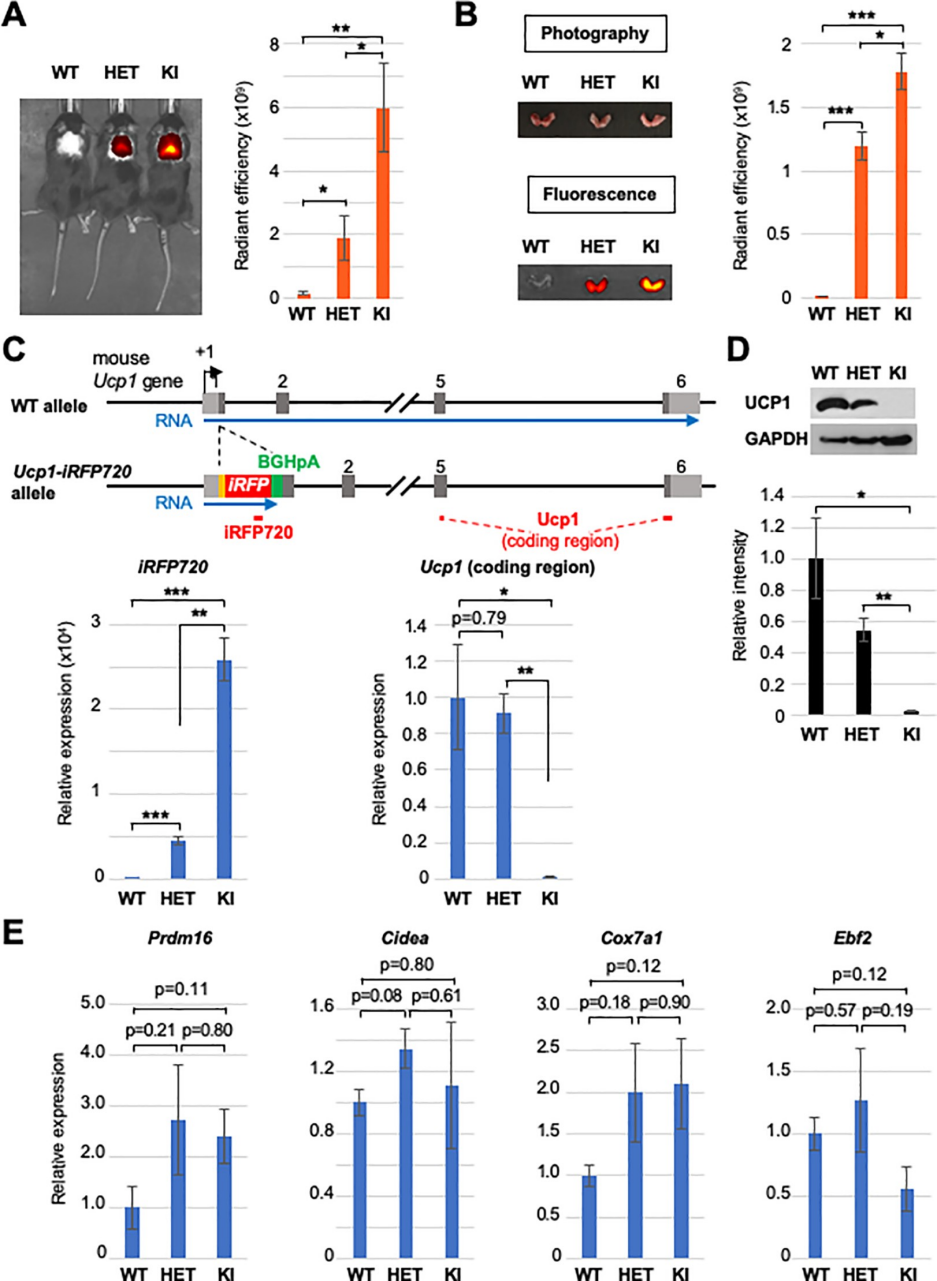
*In vivo* imaging of BAT in *Ucp1-iRFP720* KI mice. (A) *In vivo* imaging of the BATs of WT, heterozygous *Ucp1-iRFP720* KI (HET) and homozygous *Ucp1-iRFP720* KI mice (KI). Quantified fluorescence intensity for each mouse is shown in the bar graph (n = 4). ** p<0.01, * p<0.05 (B) BATs isolated from the mice used in Fig 3A. Fluorescence image of the BATs is shown in the lower panel. Quantified fluorescence intensity is shown in the bar graph (n = 4). *** p<0.001, * p<0.05 (C) Quantitative RT-PCR analysis of the *iRFP720* and *Ucp1* (coding region) in WT mice, heterozygous *Ucp1-iRFP720* KI mice and homozygous *Ucp1-iRFP720* KI mice (n = 3). The positions of the amplicons are shown as red bars. *** p<0.001, ** p<0.01, * p<0.05 (D) Expression of UCP1 in the *Ucp1-iRFP720* KI mice determined by immunoblot. GAPDH was used as a loading control. The bar graph shows the quantified intensities of UCP1 bands in the immunoblot (n = 3). ** p<0.01, * p<0.05 (E) Quantitative RT-PCR analysis of the brown adipocyte marker genes in WT mice, heterozygous *Ucp1-iRFP720* KI mice and homozygous *Ucp1-iRFP720* KI mice (n = 3).

We first analyzed the iRFP720-derived fluorescence from BAT in the dorsal interscapular region of the *Ucp1-iRFP720* KI mice. When the heterozygous and homozygous *Ucp1-iRFP720* KI mice were observed by In Vivo Imaging System (IVIS), strong fluorescence was detected in the interscapular region of both types of mice, whereas no such fluorescence was detected in WT littermates (Fig 3A). The fluorescence intensity was stronger in homozygous than heterozygous *Ucp1-iRFP720* KI mice (Fig 3A). To confirm that the fluorescence in the interscapular region of the *Ucp1-iRFP720* KI mice is indeed derived from BAT, we dissected BATs from the interscapular regions of the mice and directly observed fluorescence by IVIS. As shown in Fig 3B, BATs isolated from homozygous and heterozygous *Ucp1-iRFP720* KI mice, but not from the WT mice exhibited strong fluorescence *ex vivo*, and the fluorescence intensity correlated roughly with the number of *Ucp1-iRFP720* KI alleles, in a similar manner to the *in vivo* fluorescence images from the interscapular regions of the live mice (Fig 3A).

Analyses of mRNA expression in BAT showed that expression levels of the *iRFP720* mRNA displayed a similar pattern to that of the fluorescence levels (Fig 3A and 3C). As previously reported for the *Ucp1* knockout mice [41], the *Ucp1* mRNA levels were comparable between WT and heterozygous *Ucp1-iRFP720* KI mice, whereas homozygous *Ucp1-iRFP720* KI mice showed virtually no mRNA expression from the UCP1-coding region (Fig 3C). UCP1 protein expression was reduced to ~50% in heterozygous *Ucp1-iRFP720* KI mice, while undetectable in homozygous *Ucp1-iRFP720* KI mice (Fig 3D), as was observed for the *Ucp1* knockout mice [41]. Thus, due to the bovine growth hormone polyadenylation signal (BGHpA), transcription of the *iRFP720* mRNA did not extend into the UCP1-coding region at the *Ucp1-iRFP720* KI allele (Fig 3C). Despite the absence of UCP1 (Fig 3C and 3D), homozygous *Ucp1-iRFP720* KI mice showed no significant change in expression of other brown marker genes, such as *Prdm16*, *Cidea*, *Cox7a1*, and *Ebf2* (Fig 3E), suggesting that UCP1 loss and expression of iRFP720 do not compromise BAT development. Together, these data show that iRFP720 expressed in the BATs of the *Ucp1-iRFP720* KI mice can be imaged in live mice in a non-invasive manner.

### *In vivo* imaging of induced beige adipocytes in heterozygous *Ucp1-iRFP720* KI mice

**Fig 4:**
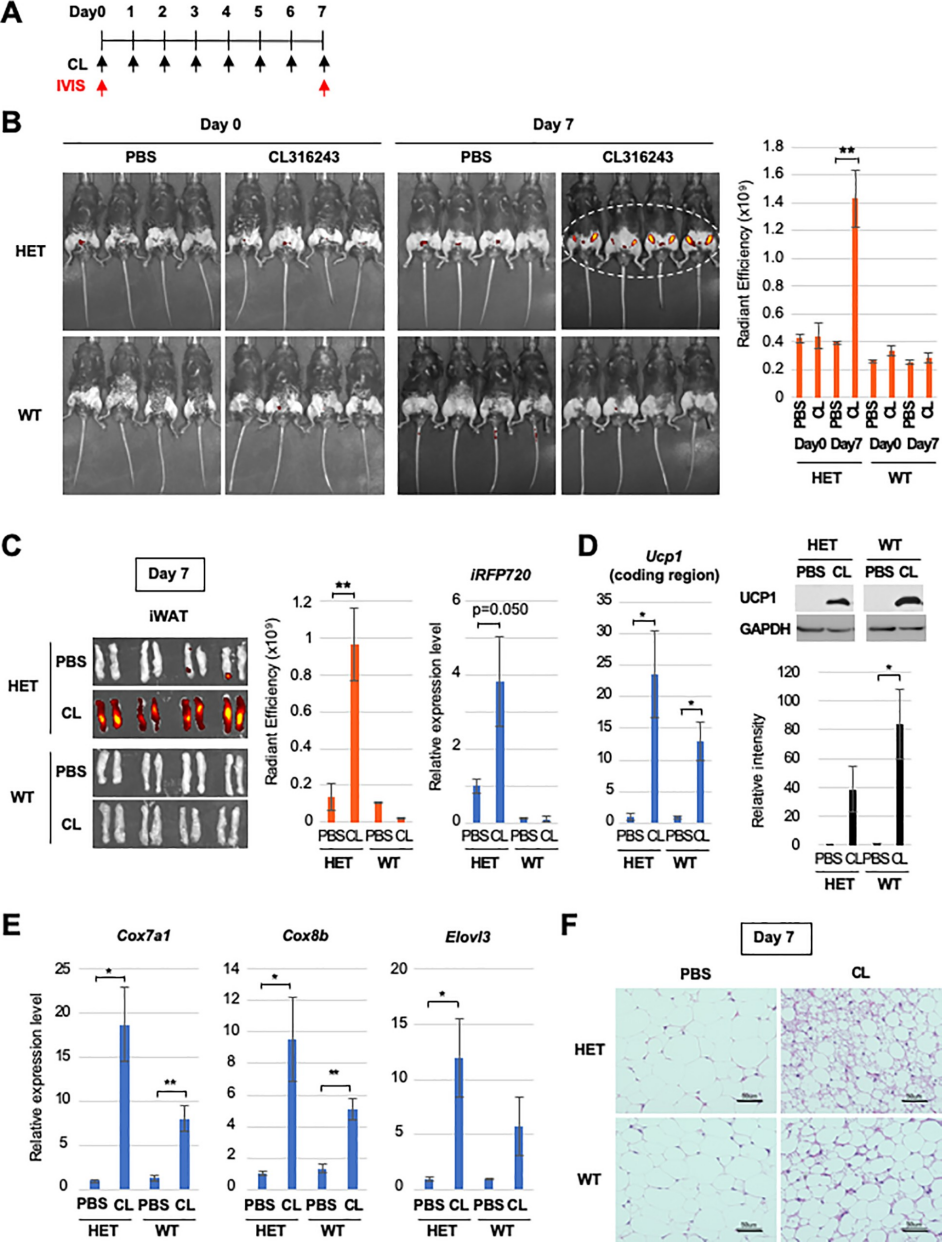
*In vivo* imaging of induced beige adipocytes. (A) Experimental scheme for beige adipocyte induction by CL316,243, which was injected intraperitoneally (1 μg/g body weight) every day for one week. Fluorescence image of the mice was captured on day 0 and day 7 by IVIS. (B) Fluorescence image of the heterozygous *Ucp1-iRFP720* KI mice and WT mice before (day 0) and after (day 7) CL316,243 treatment. Quantified fluorescence intensity is shown in the bar graph (n = 4). (C) Fluorescence image of the iWATs isolated from the mice (day 7) used in Fig 4B. The graphs show the quantification of the fluorescence (n = 4) and expression of the *iRFP720* mRNA (n = 5). ** p<0.01 (D) The expression of *Ucp1* (coding region) mRNA as well as the UCP1 protein in the iWATs used in Fig 4C (n = 3). * p<0.05 (E) Expression of the browning marker genes in the iWATs used in Fig 4C (n = 3). ** p<0.01, * p<0.05 (F) Hematoxylin and eosin staining of the iWAT sections isolated from the heterozygous *Ucp1-iRFP720* KI mice and WT mice after one-week treatment with PBS or CL316,243. Scale bar, 50 μm.

In addition to BATs, UCP1 is expressed at a lower level in beige adipocytes, which are induced in WATs by various stimuli, such as cold exposure and β3-adrenergic receptor agonists. To test if UCP1 in the induced beige adipocytes can be imaged in heterozygous *Ucp1-iRFP720* KI mice, mice were administered CL316,243, a highly selective β3-adrenergic receptor agonist, for 7 days and observed by IVIS before and after CL316,243 treatment (Fig 4A). As shown in Fig 4B, a low fluorescence signal was detected in both heterozygous *Ucp1-iRFP720* KI and WT mice before treatment with CL316,243 (Day 0). However, fluorescence was clearly detected in the inguinal regions of heterozygous *Ucp1-iRFP720* KI mice compared with control mice (treated only with PBS) after treatment with CL316,243 for 7 days (Fig 4B, upper panels, Day 7). The WT mice exhibited low levels of fluorescence even after exposure to CL316,243 for 7 days (Fig 4B, lower panels). Fluorescence intensity quantification indicated fluorescence in the inguinal regions of CL316,243-treated heterozygous *Ucp1-iRFP720* KI mice was significantly higher than that in PBS-treated mice (Fig 4B, right graph).

To confirm the source of fluorescence, iWATs were isolated and observed directly by IVIS. As shown in Fig 4C, robust fluorescence was detected in iWATs derived from heterozygous *Ucp1-iRFP720* KI mice that were treated with CL316,243. However, low fluorescence levels were observed in iWATs derived from heterozygous *Ucp1-iRFP720* KI mice treated with PBS alone, or from WT mice (Fig 4C). Consistent with greater fluorescence, the *iRFP720* mRNA was induced in iWATs from CL316,243-treated heterozygous *Ucp1-iRFP720* KI mice (Fig 4C). Moreover, the *Ucp1* mRNA and protein were induced from the intact *Ucp1* allele in heterozygous *Ucp1-iRFP720* KI mice, in a similar manner to those in WT mice (Fig 4D). In addition to *Ucp1* induction, iWAT from CL316,243-treated heterozygous *Ucp1-iRFP720* KI mice showed induction of other beige adipocyte marker genes, such as *Cox7a1*, *Cox8b*, and *Elovl3* (Fig 4E). In support of beige adipocyte induction in iWAT, histological analysis revealed clusters of smaller adipocytes with multilocular lipid droplets in HE-stained sections of iWAT from CL316,243-treated mice (Fig 4F). These data show that heterozygous *Ucp1-iRFP720* KI mice enable *in vivo* imaging of beige adipocyte induction in iWAT via iRFP720 fluorescence.

### Induction of beige-like adipocytes in homozygous *Ucp1-iRFP720* KI mice

**Fig 5:**
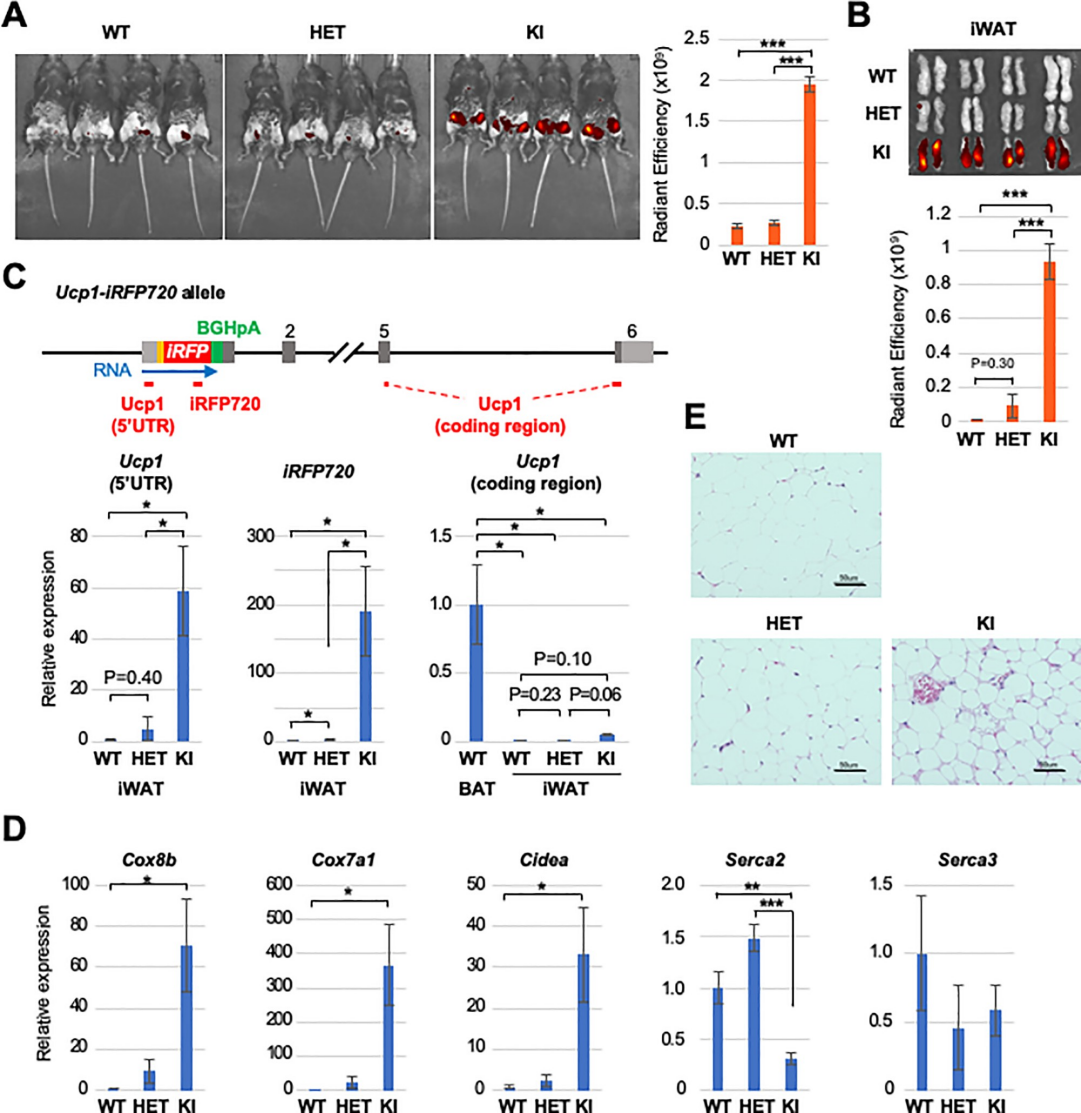
Beige-like adipocyte induction in homozygous *Ucp1-iRFP720* KI mice. (A) Fluorescence image of the inguinal regions of WT, heterozygous *Ucp1-iRFP720* KI and homozygous *Ucp1-iRFP720* KI mice. Quantified fluorescence intensity is shown in the bar graph below (n = 4). *** p<0.001 (B) Fluorescence image of the iWATs isolated from the mice shown in Fig 5A. The bar graph shows the quantified fluorescence intensity (n = 4). (C) Quantitative RT-PCR analysis of *iRFP720*, *Ucp1* 5’ untranslated region (5’ UTR) and *Ucp1* coding region in the iWATs used in Fig 5B (n = 4). The positions of the amplicons for quantitative PCR are shown as red lines below the *Ucp1-iRFP720* allele. (D) Expression of the browning marker genes (*Cox8b*, *Cox7a1*, *Cidea*) (n = 3) and UCP1-independent thermogenesis genes (*Serca2*, *Serca3*) (n = 4) in the iWATs used in Fig 5B. *** p<0.001, ** p<0.01, * p<0.05 (E) Hematoxylin and eosin staining of the iWAT sections isolated from WT, heterozygous *Ucp1-iRFP720* KI and homozygous *Ucp1-iRFP720* KI mice. Scale bar, 50 μm.

Previous studies showed that adipocytes in the inguinal region of UCP1-deficient mice adopt a brown adipocyte morphology when the mice are housed at an ambient temperature of 4~20°C [27][42][43]. To address if beige adipocytes are induced in WAT from *Ucp1* knockout mice, we generated homozygous *Ucp1-iRFP720* KI mice. When housed at the standard ambient temperature (22–24°C), which provides a mild cold stress that induces UCP1-mediated thermogenesis [44][45], homozygous *Ucp1-iRFP720* KI mice exhibited strong fluorescence in the inguinal regions, even without CL316,243 treatment (Fig 5A). Inguinal fluorescence intensity in homozygous *Ucp1-iRFP720* KI mice was comparable to that observed in CL316,243-treated heterozygous *Ucp1-iRFP720* KI mice (Figs 4B and 5A), and was significantly higher than that in heterozygous *Ucp1-iRFP720* KI or WT mice, both of which exhibited lower levels of fluorescence (Fig 5A). Indeed, iWATs isolated from homozygous *Ucp1-iRFP720* KI mice exhibited significantly stronger fluorescence than those from heterozygous *Ucp1-iRFP720* KI or WT mice (Fig 5B), indicating that fluorescence derives from inguinal iWAT. Quantitative RT-PCR analysis revealed that *iRFP720* mRNA was expressed at a high level in iWAT from homozygous *Ucp1-iRFP720* KI mice (Fig 5C). PCR analysis of the 5’ UTR region of *iRFP720* mRNA, which detects pre-mRNA as well as mRNA, also showed increased expression (Fig 5C), suggesting that the *Ucp1* gene promoter is activated in homozygous *Ucp1-iRFP720* KI mice. Due to the polyadenylation signal, the UCP1-coding region was not transcribed (Fig 5C) as observed for BAT (Fig 3C). Moreover, beige adipocyte markers, *Cox8b*, *Cox7a1*, and *Cidea*, were also induced in iWAT from homozygous *Ucp1-iRFP720* KI mice. Further, expression of *Serca2*, which is implicated in energy metabolism via calcium cycling-dependent non-canonical thermogenesis [31], was altered in homozygous *Ucp1-iRFP720* KI mice (Fig 5D). HE-stained tissue sections of iWAT showed clusters of small adipocytes with multilocular lipid droplets in homozygous *Ucp1-iRFP720* KI mice (Fig 5E). These results indicate that iWAT adipocytes from homozygous *Ucp1-iRFP720* KI mice show elevated *Ucp1* promoter activity, displaying gene expression patterns and morphology characteristics for beige adipocytes.

## Discussion

In this study we generated the *Ucp1-iRFP720* KI mice that allow *in vivo* imaging of UCP1 expression via iRFP720 fluorescence. iRFPs are near-infrared fluorescent proteins recently engineered from bacterial phytochrome photoreceptors [33]. iRFPs bind to chromophore biliverdin, which is naturally present in mammalian tissues, and exhibit bright fluorescence without an exogenous supply of the chromophore [33]. As the near-infrared light from 650 nm to 900 nm is minimally absorbed by various body components, such as hemoglobin and melanin, iRFPs are suitable for *in vivo* imaging [35]. We show here that iRFP720 expressed from the *Ucp1* locus is easily visualized in BAT of live mice. Moreover, the iRFP fluorescence is robust enough to detect lower levels of UCP1 in iWAT of live mice treated with CL316,243.

Previous studies reported bioluminescence-based *in vivo* imaging systems for UCP1 using the luciferase gene as a reporter [46][47][48]. ThermoMouse carries the *Ucp1* genomic region containing the *Ucp1* promoter-driven *luciferase-T2A-tdTomato* cassette on the Y chromosome. Luciferase expression faithfully mimics that of UCP1 in BAT and iWAT, which can be imaged in live mice upon injection of the natural substrate of luciferase, D-luciferin [46]. ThermoMouse *in vivo* imaging is restricted to males only, since the transgene is inserted randomly on the Y chromosome. Similarly, *Ucp1-2A-luciferase* knock-in mice, which carry the luciferase gene at the *Ucp1* locus, also allow *in vivo* imaging of UCP1 expression in BAT and iWAT by the luciferase reporter gene [47]. In the mice, insertion of a 2A peptide sequence in place of the stop codon of *Ucp1* inevitably attaches a 20 amino acid tag to the C-terminus of UCP1 after 2A-peptide-mediated cleavage. Although the homozygous *Ucp1-2A-luciferase* knock-in mice appear to behave normally, the effect of the added tag on UCP1-mediated thermogenesis remains unknown. Moreover, both ThermoMouse and *Ucp1-2A-luciferase* knock-in mice utilize the bioluminescence method, which requires injection of D-luciferin for *in vivo* imaging. The intensity of the luciferin signal is known to change dynamically over ~30 min, and the time course of the signal intensity can vary depending on injection methods and observed tissues [49][50]. Thus, accurate measurements of the luciferase signal in live mice is somewhat cumbersome and inconvenient.

Recently, Wang *et al* reported *Ucp1-T2A-luciferase-T2A-iRFP713* knock-in mice, which carry a dual *Ucp1* reporter, consisting of luciferase and iRFP713, inserted after the UCP1-coding region [48]. The mice supposedly enable *in vivo* imaging of UCP1 expression via both luciferase and iRFP713. Indeed, UCP1 expression is visualized *in vivo* by bioluminescence from the luciferase reporter in these mice. However, the fluorescence from iRFP713 appears to be weak and only observable in interscapular BAT of cold acclimated mice, and its use is largely restricted to *ex vivo* imaging of isolated tissues. Weak fluorescence from iRFP713 may be due to the position of the iRFP gene in the tri-cistronic construct, because expression of the fluorescent protein at the third position in tri-cistronic vectors with two 2A sequences tends to be weak [51]. In the *Ucp1-iRFP720* KI mice, the iRFP720-coding region is inserted directly at the translation initiation site of the *Ucp1* gene and expressed as a single cistron without the use of a 2A sequence. The knock-in *iRFP720* gene mimics UCP1 expression and enables *in vivo* imaging of not only BAT, but also beige adipocytes in iWAT in live, CL316,243-treated mice. The iRFP fluorescence in iWAT is almost comparable with that observed in BAT, and is significantly stronger than background autofluorescence of the liver, where bilirubin is abundant [52].

Despite its robust fluorescence, knock-in of the *iRFP* gene at the initiation codon of the *Ucp1* gene inevitably ablates *Ucp1* expression from the cognate allele. The UCP1 level in heterozygous *Ucp1-iRFP720* KI mice is reduced to ~50% of that observed in WT mice, and UCP1 was undetectable in homozygous *Ucp1-iRFP720* KI mice (Fig 3D). However, given that heterozygous *Ucp1* knockout mice exhibit minimal phenotypic characteristics [41], heterozygous *Ucp1-iRFP720* KI mice are presumed to be phenotypically normal. Since heterozygous *Ucp1-iRFP720* KI mice express iRFP720 from the knock-in allele, and UCP1 from the other allele, it is possible to image UCP1 expression *in viv*o via iRFP720 fluorescence, and to identify brown and beige adipocytes in isolated tissue samples via UCP1 immunostaining.

In contrast, homozygous KI mice serve as a UCP1-deficient mouse model, with the added advantage of imaging and identifying cells that would otherwise express UCP1. Previous studies showed that UCP1-deficient mice are sensitive to cold, but paradoxically more resistant to diet-induced obesity [42]. This resistance to obesity occurs during cold stress (4~20°C), but is absent at thermoneutrality (27°C), suggesting the presence of alternative UCP1-independent non-shivering thermogenesis that is less efficient than UCP1-dependent thermogenesis. iWAT from UCP1-deficient mice housed at 4~20°C exhibit the appearance of beige-like adipocytes, suggesting a probable location for alternative thermogenesis [27][42][43], which are also observed in *Ucp1-iRFP720* KI mice (Fig 5). Given the recent identification of futile cycles in beige adipocytes, such as creatine-dependent ADP/ATP substrate cycling [28][29][30] and calcium cycling [31], it would be interesting to investigate the expansion of beige adipocytes in iWAT and the rise of alternative thermogenesis in these adipocytes. Elevated activation of the *Ucp1* gene promoter in homozygous *Ucp1-iRFP720* KI mice housed under standard conditions indicates possible auto-regulatory pathways, which are constitutively activated by UCP1 loss. The mice described in this study should be particularly useful for identifying such pathways that lead to beige adipocyte induction and alternative non-shivering thermogenesis in iWAT *in vivo*. A recent study using single-cell RNA sequencing revealed a developmental hierarchy of adipocyte progenitors [53]. A similar method can be applied to stromal vascular fraction cells in iWAT from *Ucp1-iRFP720* KI mice to investigate how iRFP-positive and–negative adipocytes arise from mesenchymal progenitors and where their developmental pathways diverge. In addition to stromal vascular fraction cells, mature adipocytes may be sorted by using iRFP720 as a reporter in a flow cytometry method [54]. The isolated iRFP-positive and–negative mature adipocytes may allow analysis of distinct metabolic and thermogenic properties of these two types of adipocytes. In conclusion, *Ucp1-iRFP720* KI mice described here serve as a convenient mouse model for *in vivo* imaging and *in vitro* analyses of UCP1-expressing adipocytes in both BAT and iWAT.

## Supporting information

S1 Table Primers for genomic PCR. (PDF)

S2 Table Primers for qPCR. (PDF)

S1 Raw images (PDF)

## Abbreviations

UCP1: Uncoupling protein 1
iRFP: near-infrared fluorescent protein
BAT: brown adipose tissue
WAT: white adipose tissue
NIR: near-infrared
BGHpA: bovine growth hormone polyadenylation signal
WT: wild-type
