# Supplementary material for: Non-invasive in vivo imaging of UCP1 expression in live mice via near-infrared fluorescent protein iRFP720
Source: PLoS One. 2019 Nov 15;14(11):e0225213. doi: 10.1371/journal.pone.0225213 (PMC6857924; doi:10.1371/journal.pone.0225213)
Supplement: S1 Table — (PDF) [file pone.0225213.s001.pdf]

S1 Table

Primers for genomic PCR

Primer	Sequence
a	ACGATGAGCCGATCCATATC
b	GCAGGCCTAGTTTTGACTCG
c	CCAAGTTGTTCCTGGTTGCTCTCAATAA
d	AATGATCCGTACCGGGTTGATGGTATAG
e	TCCACAGGGGCCACCATGGCGGAAGGATCCGTCGCCAGG
f	GCCCAATACACAAGCCCTAACACCATTAGATG
g	GCCTGAAGAGATTGCCCAAGTTCTCACAGAC
h	CACGCTCACCGGCTCCAGATTTATC
i	CAGGAGGCAGTCAAGAGCAGCTTC
j	AGTTCATCAAGCCCATCCTG
k	GAAGTTTCTGTTGGCGAAGC
