# Supplementary material for: Non-invasive in vivo imaging of UCP1 expression in live mice via near-infrared fluorescent protein iRFP720
Source: PLoS One. 2019 Nov 15;14(11):e0225213. doi: 10.1371/journal.pone.0225213 (PMC6857924; doi:10.1371/journal.pone.0225213)
Supplement: S2 Table — (PDF) [file pone.0225213.s002.pdf]

S2 Table

Primers for qPCR

Gene	Sequence
iRFP720	CGGCCGATTGATCTTAGCTTC
	GCGCATGAACTCCAGATGGT
Ucp1 (coding region)	GGACAGTACCCAAGCGTACCA
	CGCAGAAAAGAAGCCACAAAC
Prdm16	TGCGAAGGTGTCCAAACTGA
	GCCCAGAAGCAGCTACACACT
Cox7a1	TGGCAGAGAAGCAGAAGCTCTT
	CCCCCGCCTTTCAAGTGT
Cox8b	TTCCCAAAGCCCATGTCTCT
	GCTCCACGGCGGAAGTG
Elovl3	GGAGGAGTACTGGGTAAGCTCATTT
	GGCCAACAACGATGAGCAA
Ucp1 (5' UTR)	GAAGGGACGCTCACCTTTG
	CTAGGTAGTGCCAGTGCAGA
Cidea	ACAGAAATGGACACCGGGTAGT
	TCCCGATTTCTTTGGTTGCT
Nono	TGCTCCTGTGCCACCTGGTACTC
	CCGGAGCTGGACGGTTGAATGC
Ebf2	TGACAAAGAGCAAGGCAATG
	TGGTGACAGAGTCGATGAGC
Dio2	CAGTGTGGTGCACGTCTCCAATC
	TGAACCAAAGTTGACCACCAG
Serca2	TCGACAGGACAGAAAGAGTGTG
	AAACTGAATTCAACTCACCAGC
Serca3	GGAGCAGTTTGAGGACCTCTT
	GGCCACGAGAATTAGCATGATG
