# Supplementary figures and images for: Non-invasive in vivo imaging of UCP1 expression in live mice via near-infrared fluorescent protein iRFP720
Source: PLoS One. 2019 Nov 15;14(11):e0225213. doi: 10.1371/journal.pone.0225213 (PMC6857924; doi:10.1371/journal.pone.0225213)

Figure 2B

a+b

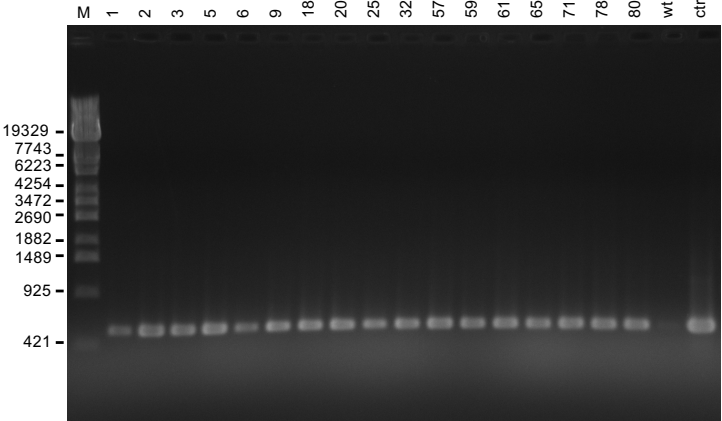

Figure 2C

h+i

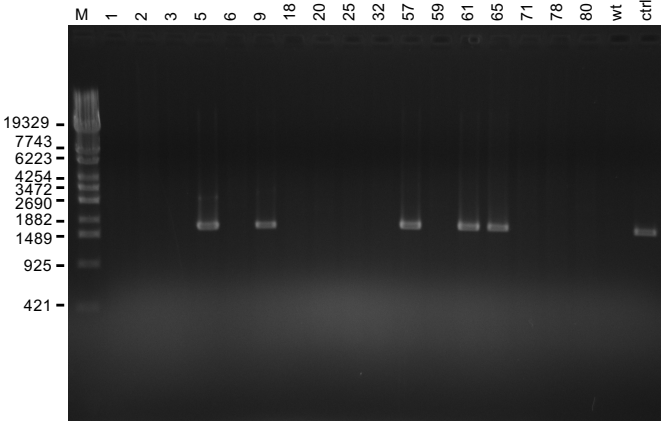

c+d

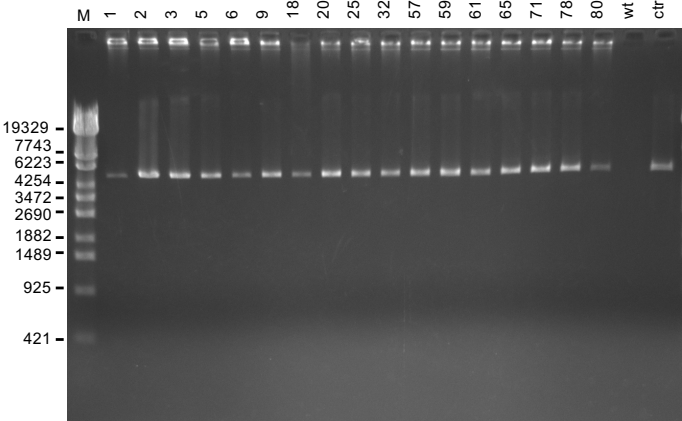

j+k

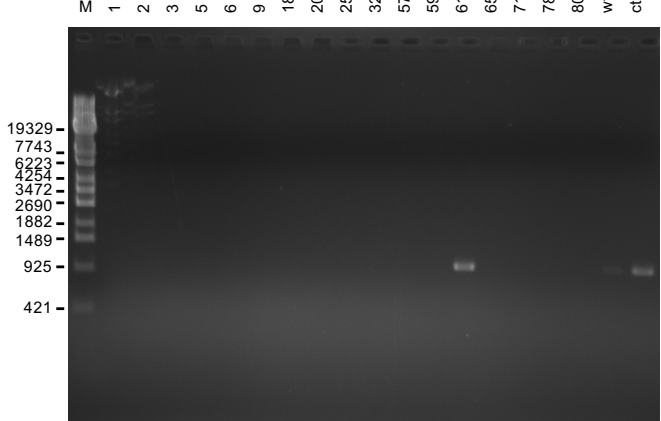

e+f

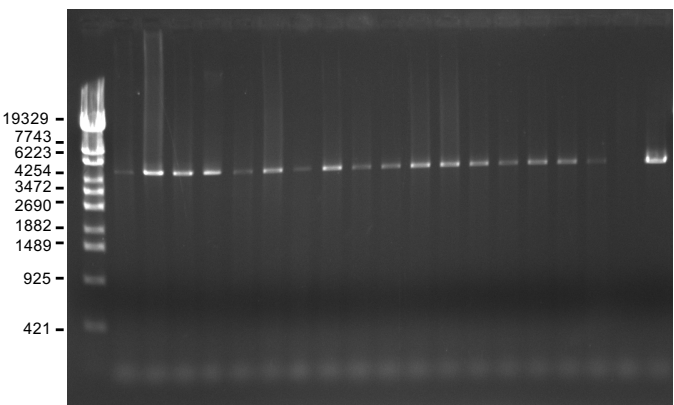

g+f

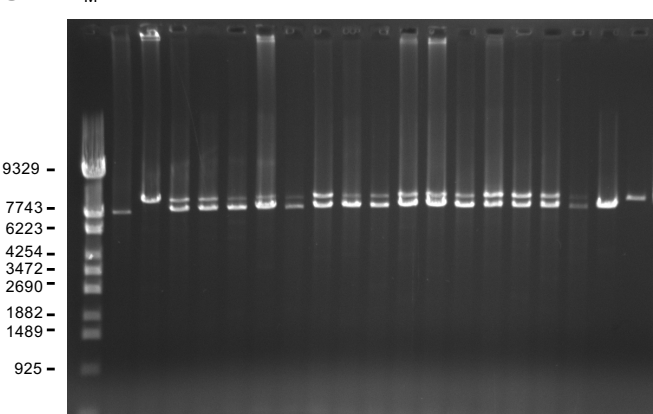

Figure 2D

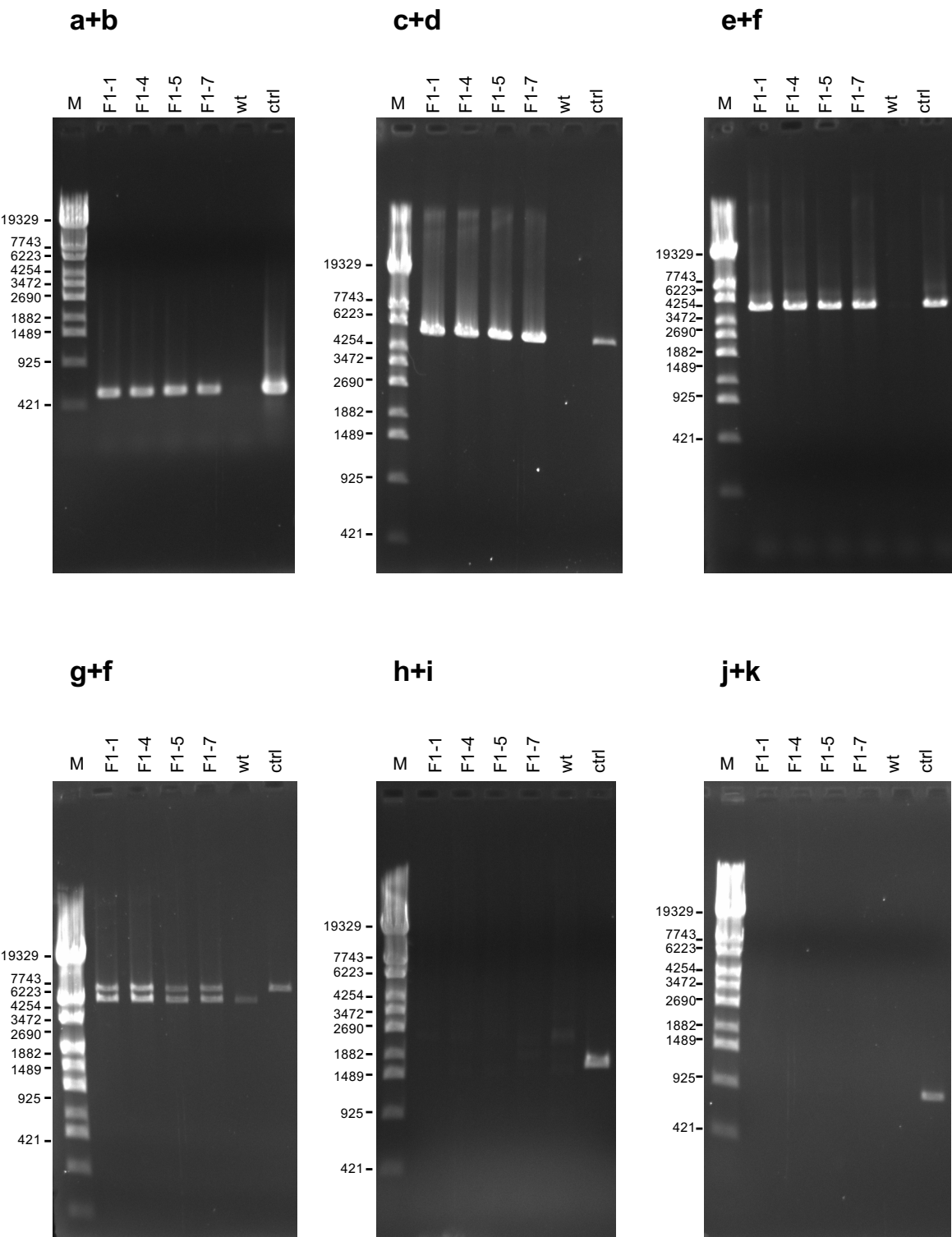

**Figure 3D**

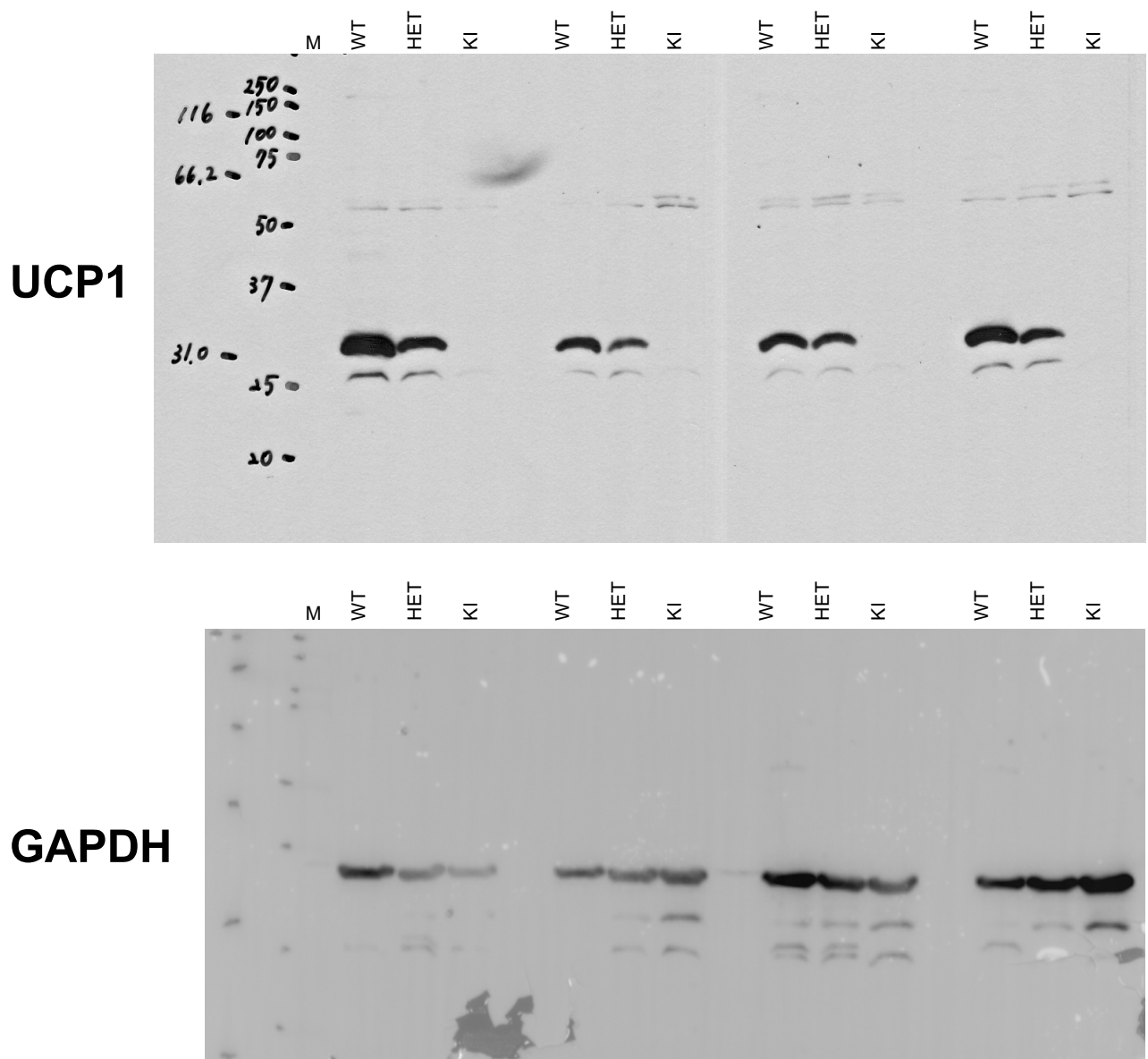

### Figure 4D

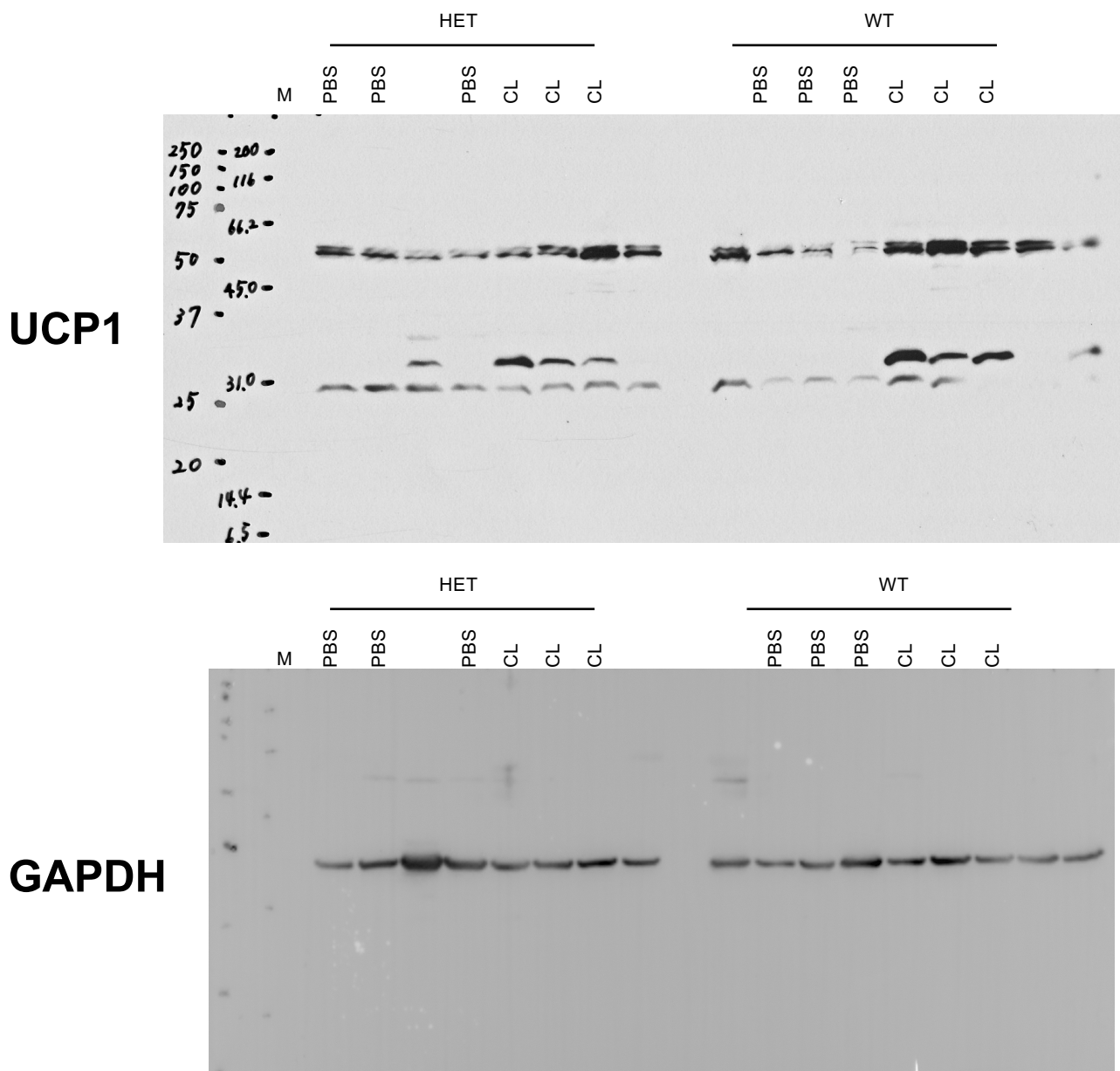

Supplement: S1 Raw images — (PDF) [file pone.0225213.s003.pdf]
